# Carotenoid biosynthesis and overproduction in *Corynebacterium glutamicum*

**DOI:** 10.1186/1471-2180-12-198

**Published:** 2012-09-10

**Authors:** Sabine A E Heider, Petra Peters-Wendisch, Volker F Wendisch

**Affiliations:** 1 Chair of Genetics of Prokaryotes, Faculty of Biology & CeBiTec, University of Bielefeld, P.O. Box 100131, D-33501, Bielefeld, Germany

**Keywords:** *Corynebacterium*, Carotenoid production, Lycopene production, Phytoene synthase, Phytoene desaturase, Lycopene elongase, Carotenoid epsilon-cyclase, Decaprenoxanthin

## Abstract

**Background:**

*Corynebacterium glutamicum* contains the glycosylated C50 carotenoid decaprenoxanthin as yellow pigment. Starting from isopentenyl pyrophosphate, which is generated in the non-mevalonate pathway, decaprenoxanthin is synthesized via the intermediates farnesyl pyrophosphate, geranylgeranyl pyrophosphate, lycopene and flavuxanthin.

**Results:**

Here, we showed that the genes of the carotenoid gene cluster *crtE-cg0722-crtBIY*_*e*_*Y*_*f*_*Eb* are co-transcribed and characterized defined gene deletion mutants. Gene deletion analysis revealed that c*rtI, crtEb,* and *crtY*_*e*_*Y*_*f*_, respectively, code for the only phytoene desaturase, lycopene elongase, and carotenoid C45/C50 ɛ-cyclase, respectively. However, the genome of *C. glutamicum* also encodes a second carotenoid gene cluster comprising *crtB2I2-1/2* shown to be co-transcribed, as well. Ectopic expression of *crtB2* could compensate for the lack of phytoene synthase CrtB in *C. glutamicum* Δ*crtB*, thus, *C. glutamicum* possesses two functional phytoene synthases, namely CrtB and CrtB2. Genetic evidence for a *crtI2-1/2* encoded phytoene desaturase could not be obtained since plasmid-borne expression of *crtI2-1/2* did not compensate for the lack of phytoene desaturase CrtI in *C. glutamicum* Δ*crtI*. The potential of *C. glutamicum* to overproduce carotenoids was estimated with lycopene as example. Deletion of the gene *crtEb* prevented conversion of lycopene to decaprenoxanthin and entailed accumulation of lycopene to 0.03 ± 0.01 mg/g cell dry weight (CDW). When the genes *crtE, crtB* and *crtI* for conversion of geranylgeranyl pyrophosphate to lycopene were overexpressed in *C. glutamicum* Δ*crtEb* intensely red-pigmented cells and an 80 fold increased lycopene content of 2.4 ± 0.3 mg/g CDW were obtained*.*

**Conclusion:**

*C. glutamicum* possesses a certain degree of redundancy in the biosynthesis of the C50 carotenoid decaprenoxanthin as it possesses two functional phytoene synthase genes. Already metabolic engineering of only the terminal reactions leading to lycopene resulted in considerable lycopene production indicating that *C. glutamicum* may serve as a potential host for carotenoid production.

## Background

Carotenoids are yellow to red colored pigments originating from the terpenoid biosynthetic pathway. They are very abundant in plants and microorganisms, where they can have diverse functions such as photo protection or light harvesting molecules or as membrane stabilizers [1,2]. In the biosynthetic pathways of certain hormones (like retinoic acid, a hormone regulating the epidermal growth of mammals) they serve as precursors [3]. Carotenoids are also proposed to prevent cancer and reduce the risk of cardiovascular and Alzheimer disease due to their antioxidative properties [4-6]. Traditionally, terpenoids have been used in the feed, food and nutraceutical industries [1]. As the large-scale chemical synthesis of terpenoids is often difficult and/or costly due to their structural complexity [7] and as their isolation from natural sources usually does not yield sufficient quantities [8], microbial production processes offer a promising alternative.

Carotenoids are derived from the universal precursor isopentenyl pyrophosphate (IPP) and its isomer dimethylallyl pyrophosphate (DMPP) [9]. Enhancing cellular metabolic flux toward IPP and DMAPP is one strategy to improve rates and yield of microbial isoprenoid production [10,11]. There are two independent pathways leading to IPP: the mevalonic acid (MVA) pathway and the methylerythritol phosphate (MEP) pathway. The MVA pathway is found in eukaryotes (mammals, fungi, in the cytoplasm of plant cells), archaea, and a limited number of bacteria. Most bacteria as well as plant plastides synthesize IPP through the MEP pathway [1,12,13]. The MVA pathway requires acetyl-CoA as the primary educt, whereas the MEP pathway starts by condensation of pyruvate and glyceraldehyde 3-phosphate (GAP) [14,15].

*Corynebacterium glutamicum* is used commercially for the annual production of more than 3,000,000 tons of amino acids (Ajinomoto, Food Products Business. Available from World Wide Web: http://www.ajinomoto.com/ir/pdf/Food-Oct2010.pdf. 2010, cited 20 April 2012). The predominant carotenoids in *C. glutamicum* are the C50-terpene decaprenoxanthin and its glucosides [16]. To date, only three different C50 carotenoid biosynthetic pathways have been described: the biosynthetic pathways of the ɛ-cyclic C50 carotenoid decaprenoxanthin in *C. glutamicum*[17,18], the β-cyclic C50 carotenoid C.p. 450 in *Dietzia* sp. CQ4 [19] and the γ-cyclic C50 carotenoid sarcinaxanthin in *Micrococcus luteus* NCTC2665 [20]. In addition, only a few other corynebacteria have been identified to contain carotenoid pigments i.e. *C. michiganense*[21], *C*. *erythrogenes*[22], *C. fascians*[23] and *C. poinsettiae*[24]. *C. poinsettiae* (*Curtobacterium flaccumfaciens*) e.g. is known to produce the C50 carotenoids bacterioruberin, bisanhydrobacterioruberin and C.p. 450 [2]. The genome of *C. glutamicum* encodes the enzymes of the MEP pathway [2,25]. Based on transposon mutant analysis and biochemical evidence *C. glutamicum* possesses a carotenogenic gene cluster encoding the responsible enzymes for the entire decaprenoxanthin biosynthesis starting from DMPP [17,18]. The immediate precursors of C30 and C40 carotenoids, farnesyl pyrophosphate (FPP, C15) and geranylgeranyl pyrophosphate (GGPP, C20), are generated from DMPP by prenyl transferase CrtE [18]. Subsequently, phytoene synthase (CrtB) condenses two GGPP molecules yielding the colorless carotenoid phytoene. Four subsequent desaturation reactions by phytoene desaturase (CrtI) yield the red-colored lycopene [17,18]. The elongation of lycopene with DMPP to the acyclic C50 carotenoid flavuxanthin is catalyzed by the *crtEb* gene product lycopene elongase. The cyclization of flavuxanthin to decaprenoxanthin is catalyzed by heterodimeric carotenoid -ɛ-cyclase, encoded by *crtY*_*e*_ and *crtY*_*f*_[16,20,26]. While mono- and diglucosylated decaprenoxanthin can be found in *C. glutamicum*, the genes and enzymes for glucosylation of decaprenoxanthin are still unknown [20].

In this study, gene-directed deletion mutagenesis was employed to decipher the functions of the genes present in the main carotenogenic gene cluster of *C. glutamicum* and in a second cluster encoding putative phytoene synthase and phytoene desaturase paralogs. Moreover, the potential of *C. glutamicum* to produce carotenoids was estimated by metabolic engineering of the conversion of GGPP to lycopene.

## Results

### Bioinformatical analysis of the carotenogenic genes

**Figure 1:**
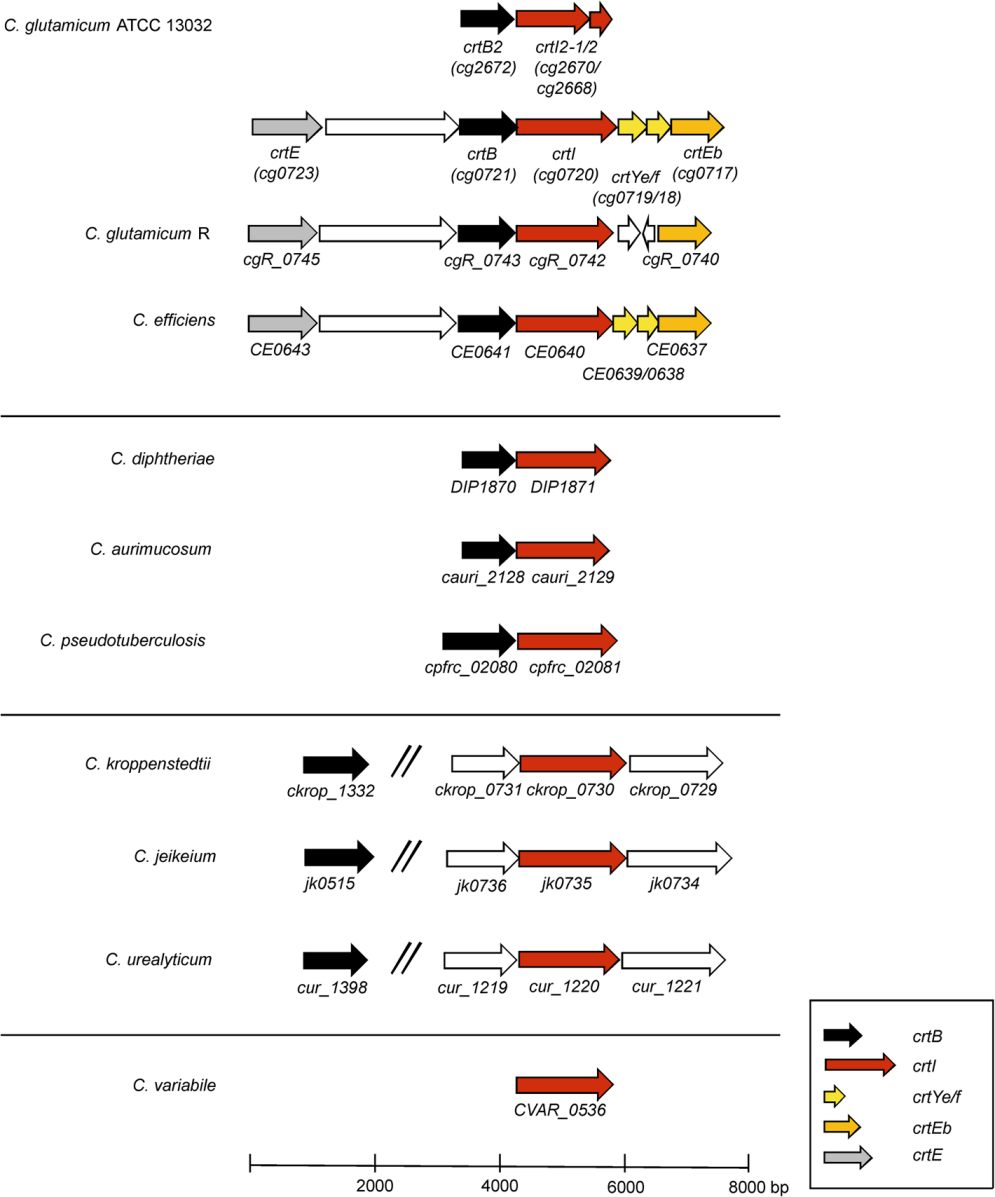
**Genomic organization of the putative and characterized carotenogenic genes in different corynebacteria**.

**Figure 2:**
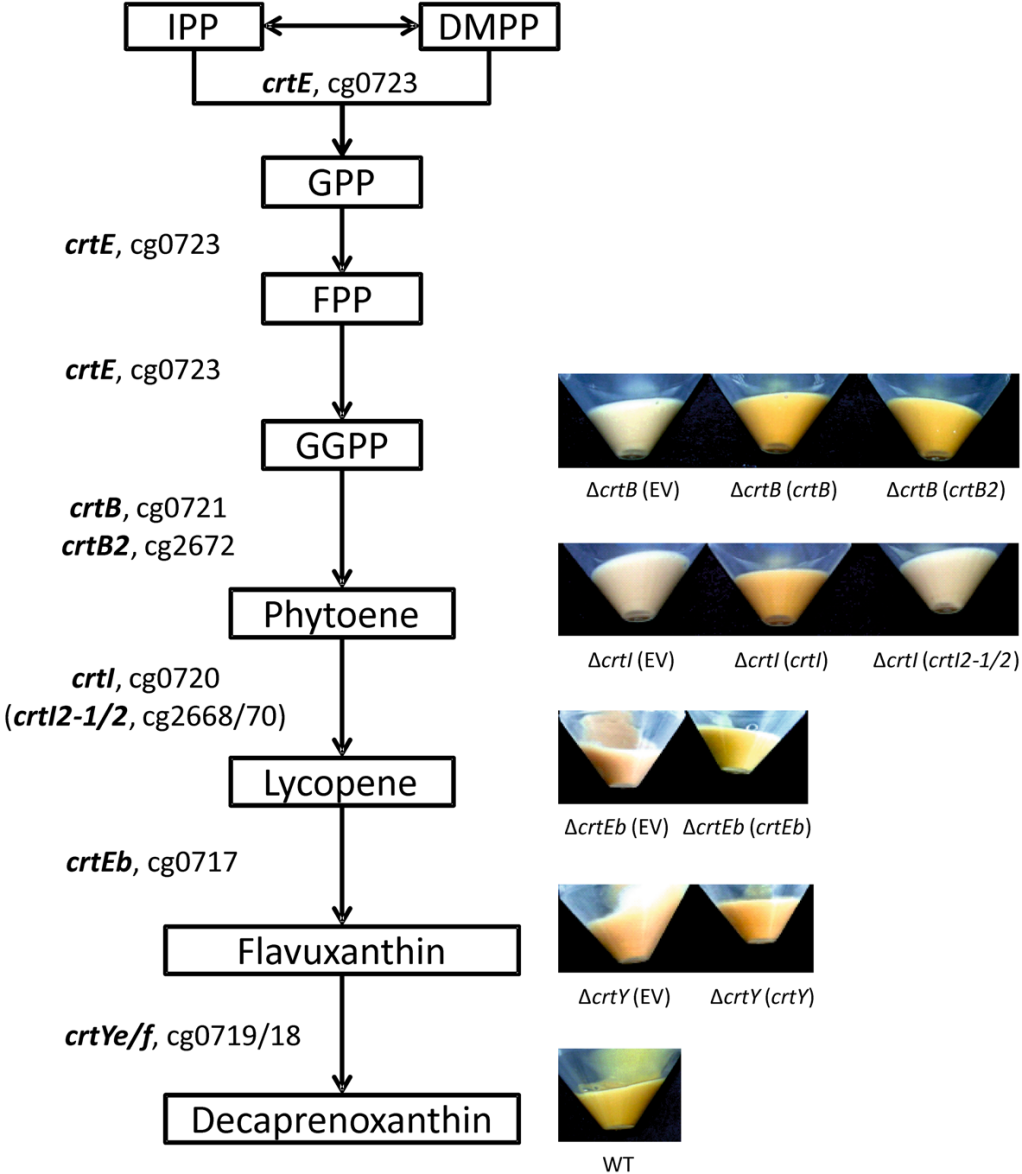
**Carotenoid biosynthesis in *C. glutamicum *ATCC 13032 and gene deletion and complementation analysis of carotenogenic genes.** Cell pellets of *C. glutamicum* deletion mutants lacking one of the carotenogenic genes *crtB*, *crtI*, *crtEb* or *crtY*_*e*_*Y*_*f*_ and the wild type and the corresponding complemented strains (right, EV: empty vector). The cells were grown in 50 ml CGXII medium with 100 mM glucose, inoculated to an OD_600_ of 1 with a BHI overnight culture. The overexpression was induced at the beginning of the cultivation with 1 mM IPTG.

The genome of *C. glutamicum* ATCC 13032 (wild type; WT) encodes genes showing homology to carotenoid biosynthesis genes in two gene clusters that are separated by almost 2 Mbp. The larger cluster is composed of seven genes, *crtE* (cg0723), cg0722 (encoding a putative membrane protein), *crtB* (cg0721), *crtI* (cg0720), *crtY*_*e*_*, crtY*_*f*_ (cg01719/18) and *crtEb* (cg0717) (Figure 1 and 2). The second cluster consists of a gene putatively encoding phytoene synthase (here named *crtB2*, cg2672) and two genes with similarity to an N-terminal fragment (*crtI2-1*, cg2670) and a C-terminal fragment (*crtI2-2*, cg2668) of phytoene desaturase/dehydrogenase (Figure 1).

The cluster *crtB2/crtI2-1/crtI2-2* has not yet been analyzed. While CrtB and CrtB2 share 49% identity, CrtI2-1 shares 49% identical amino acids with the 364 N-terminal amino acids of CrtI and CrtI2-2 63% identical amino acids with the 104 C-terminal amino acids of CrtI. Thus, it is not clear whether CrtI2-1 and CrtI2-2 function as a two-subunit phytoene desaturase or whether a frameshift mutation disrupted the gene downstream of *crtB2*.

A comparison of the sequenced genomes of corynebacteria (Figure 1, Additional file 1: Table S1) revealed that *C. glutamicum* WT is the only species possessing two *crtB* and *crtI* like genes, while the organization of the large gene cluster is comparable in *C. glutamicum* WT, *C. glutamicum* R (and ATCC 14067 and S9114) and *C. efficiens* YS-314. In *C. glutamicum* R, no *crtY*_*e*_*Y*_*f*_ is annotated as likely a G- > T mutation at position 814478 of the *C. glutamicum* R genome altered the start codon of an open reading frame coding for a protein with 99% amino acid identity to *crtY*_*e*_*Y*_*f*_ of *C. glutamicum* WT to a leucine codon.

A second group of corynebacterial species (e.g. *C. diphteriae*, *C. aurimucosum* and *C. pseudotuberculosis)* only possess the clustered genes *crtB* and *crtI* (50 to 55% amino acid identity to the *C. glutamicum* enzymes; Additional file 1: Table S1). An intermediate situation is found in *C. lipophiloflavum*, which possesses a gene cluster with *crtB*, *crtI*, *crtY*_*e/f*_ and *crtEb,* as well as in *C. genitalium* possessing *crtB*, *crtI* and *crtY*_*e/f*_ but lacking *crtEb* (Additional file 1: Table S1). Members of a third group (*C. kroppenstedtii*, *C. jeikeium*, *C. urealyticum* as examples) also lack *crtY*_*e/f*_ and *crtEb* orthologs, but possess *crtB* and *crtI*, however not clustered. Although the overall amino acid sequence identities of the *crtB* and *crtI* gene products are below 50% as compared to the respective CrtB and CrtI from *C. glutamcium* WT, their domain structure includes the *crtI* domain (TIGR02734) as well as an N-terminal NAD(P)-binding Rossmann-like domain (NCBI Domain structure). As an exception, *C. variabile* only possesses CrtI with an amino acid identity to CrtI from *C. glutamicum* WT of 58%.

The phylogeny of the *crtI* gene product (Additional file 2: Figure S1), which is present in all analysed corynebacteria, is congruent to the grouping of cornyebacterial species with respect to occurrence and clustering of *crt* genes as shown in Figure 1 and Additional file 1: Table S1.

### Analysis of the transcriptional organization of the carotenogenic gene clusters

**Figure 3:**
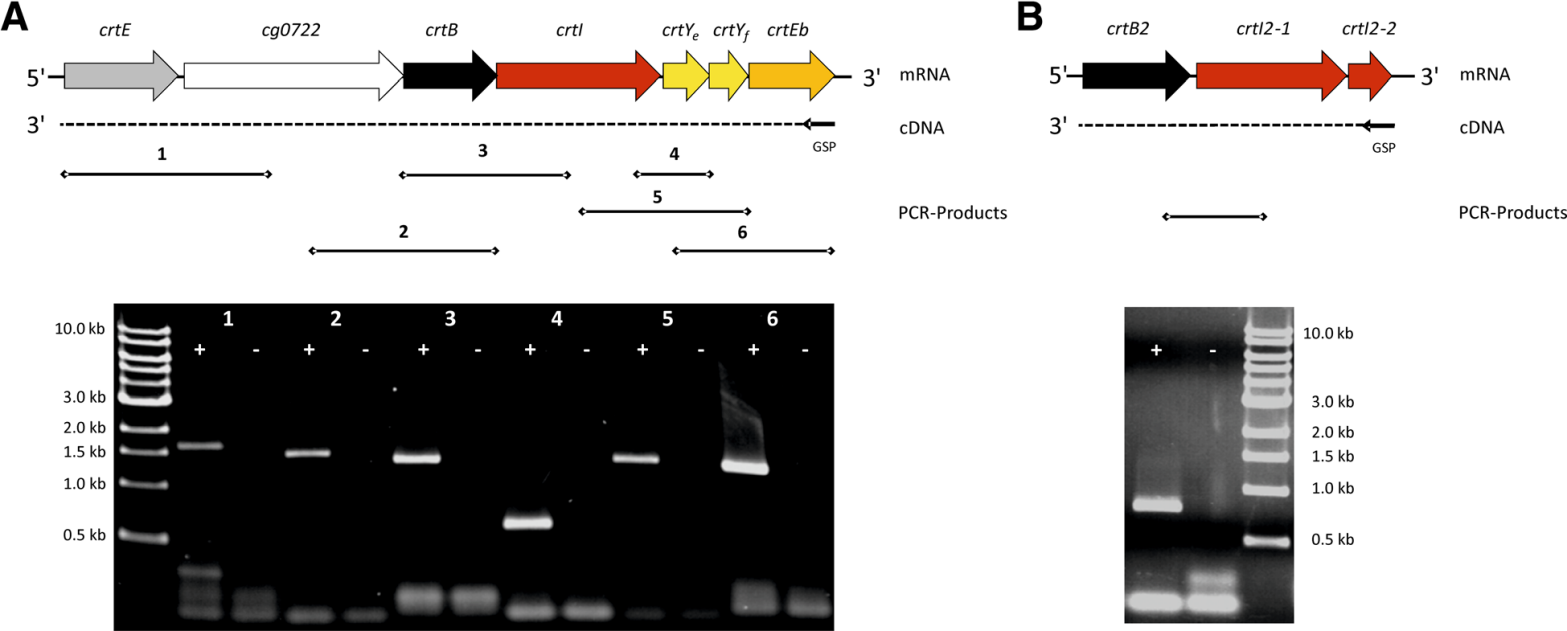
**Transcriptional organization of the carotenogenic gene clusters in *C. glutamicum *ATCC 13032.** The schemes show the respective *crt* loci (*crtE-cg0722-crtBIY*_*e*_*Y*_*f*_*Eb* (**A**); *crtB2I2-1/2* (**B**)) in *C. glutamicum* and the RT-PCR reactions used to determine co-transcription of the *crt* gene clusters. RNA from *C. glutamicum* WT was transcribed into cDNA with gene specific primers of the last gene in 3’ direction of the predicted operons. Subsequently, cDNAs were used as templates for six different PCR reactions for *crtB*, *crtI*, *crtEb* and *crtY*_*e*_*Y*_*f*_ (labeled 1 – 6 (**A**)) and one PCR reaction for *crtB2, crtI2-1* and *crtI2-2* (**B**). Reactions labeled (−) represent controls confirming the absence of DNA in the RNA preparation. The reactions were identical to the PCR reactions as shown in the lanes labeled as (+) except that reverse transcriptase was omitted in the cDNA reactions.

Annotation of the carotenogenic gene cluster of the *C. glutamicum* WT for the biosynthesis of decaprenoxanthin from the precursor GGPP suggests co-transcription of *crtB*, *crtI*, *crtY*_*e*_ and *crtY*_*f*_ and *crtEb*, while the upstream GGPP synthase gene *crtE* appears to be monocistronic. To characterize the transcriptional organization of this cluster RT-PCR experiments have been carried out. PCR analysis of cDNA synthesized from total RNA of the *C. glutamicum* WT using primer *crtEb*-rv (see Additional file 3: Table S2) revealed that the entire gene cluster is co-transcribed since fragments overlapping adjacent genes could be amplified in each case. A cDNA preparation without the addition of reverse transcriptase served as a negative control (Figure 3).

Similarly, RT-PCR analysis of the small gene cluster revealed that *crtB2*, *crtI2-1* and *crtI2-2* are co-transcribed. Figure 3B displays the amplificate of a fragment overlapping *crtB2* and *crtI2-1* based on cDNA generated by reverse transcription using the *crtI*-rv primer.

To determine the transcriptional start point (TSP) of *crtE* and *crtB2*, respectively, RNA was isolated from *C. glutamicum* WT grown in LB complex medium. By use of 5’ RACE_PCR, the TSP of *crtE* was identified as a guanosine 114 nucleotides upstream of the first nucleotide of the ATG start codon. The three most conserved nucleotides of the consensus −10 hexamer of *C. glutamicum* promoters [27] can be found in the −15 to −10 region. The −39 to −34 region contains a sequence motif sharing four identical nucleotides to −35 consensus. The TSP of *crtB2* was determined as a guanosin thirteen nucleotides upstream of the first nucleotide of the start codon GTG. The hexamer TAAAGT at position −13 to −8 relative to the TSP matches the three most conserved bases of the TANANT consensus sequence of the −10 region of *C. glutamicum* promoters [27]. At position −32 to −27 the hexamer TTGTCT was found, which resembles the key recognition motif for the −35 region of *C. glutamicum* promoters TTGNCA [27].

### Gene deletion and complementation analysis of the carotenogenic gene clusters in *C. glutamicum*

Gene-directed deletion mutants of *C. glutamicum* WT lacking *crtB, crtI, crtEb,* or *crtY*_*e*_*Y*_*f*_ were constructed and characterized regarding carotenoid production. Besides the single deletion mutants, strain *C. glutamicum* ΔΔ lacking *crtB, crtI, crtEb,* and *crtY*_*e*_*Y*_*f*_ as well as the putative paralogs *crtB2, crtI2-1* and *crtI2-2* was constructed. All strains showed growth rates of about 0.35 h^-1^ in CGXII minimal medium with 100 mM glucose as carbon source. Thus, growth was comparable to *C. glutamicum* WT. However, pigment accumulation differed between the various strains (Figure 2). The different composition of carotenoids in the cell extracts could be demonstrated by HPLC analyses (Additional file 4: Figure S2, Additional file 5: Figure S3, Additional file 6: S4 and data not shown). The spectrophotometric analysis of the methanolic cell extracts of the *C. glutamicum* WT showed the characteristic absorption maxima at 415, 440, and 470 nm for the yellow decaprenoxanthin, whereas the spectra of the pale red-colored *C. glutamicum* strains Δ*crtEb* and Δ*crtY* showed absorption maxima at 445, 470 and 500 nm (Additional file 4: Figure S2).

The multiple deletion strain *C. glutamicum* ΔΔ (Additional file 3: Table S2) was used for stepwise reconstruction of the decaprenoxanthin biosynthetic pathway. Expression of *crtB* and *crtI* in the white strain *C. glutamicum* ΔΔ entailed a pale pink cell color and accumulation of lycopene was observed in cell extracts. Additional expression of *crtEb* entailed an orange cell color and accumulation of flavuxanthin. When *crtY*_*e*_*Y*_*f*_ was expressed additionally, a color comparable to that of the wild type was observed and the HPLC chromatograms of the cell extracts were comparable to those of the wild type. Thus, expression of *crtB, crtI, crtEb, crtY*_*e*_ and *crtY*_*f*_ in the multiple deletion strain was sufficient to allow for decaprenoxanthin biosynthesis.

This finding was supported by analysis of the single gene deletion strains. Each deletion mutant could be complemented by ectopic expression of the respective gene deleted in the chromosome (Figure 2). The mutant Δ*crtY* lacking the final reaction in the synthesis of decaprenoxanthin, i.e. introduction of two ɛ-ionone groups into the acyclic flavuxanthin catalyzed by gene products of *crtY*_*e*_*Y*_*f*_, accumulated flavuxanthin and exhibited a pale orange to red color. In the absence of the penultimate enzyme reaction of decaprenoxanthin biosynthesis, i.e. prenylation of lycopene to flavuxanthin by lycopene elongase, in the mutant Δ*crtEb*, lycopene accumulated and neither flavuxanthin nor decaprenoxanthin were observed (HPLC analysis of cell extracts not shown). Accordingly, mutants Δ*crtB* lacking phytoene synthase and Δ*crtI* lacking phytoene desaturase showed white cell color and Δ*crtI* accumulated phytoene, which absorbs light at wavelengths below 300 nm. Taken together, our gene deletion and complementation analysis corroborates previous biochemical and transposon mutagenesis data and results from heterologous gene expression regarding the functions of the enzymes encoded by *crtB, crtI, crtEb, crtY*_*e*_ and *crtY*_*f*_.

The function of the putative *crtB* paralogous gene *crtB2* and of the putative *crtI* paralogous genes *crtI2-1* and *crtI2-2* has not yet been analyzed. As hardly any phytoene was detectable in Δ*crtB*, but faint quantities of other carotenogenic intermediates were observed, CrtB appears to be the major phytoene synthase active under the chosen conditions. Similarly, the lack of the red chromophore lycopene in Δ*crtI* indicated that CrtI is the only active phytoene desaturase. By contrast, a deletion mutant lacking the paralogous genes *crtB2, crtI2-1* and *crtI2-2* showed the same yellow phenotype as *C. glutamicum* WT and the cell extracts showed the identical elution pattern in the HPLC analysis. Moreover, deletion mutants lacking *crtB2I2-1I2-2* and either *crtB* or *crtI* grew like wild type and showed the same white phenotype as the *crtB* and *crtI* single deletion mutants. Thus, the paralogous genes annotated as *crtB2* and *crtI2-1* and *crtI2-2* are either not functional or not expressed (enough) under the chosen conditions.

Complementation analysis of the deletion mutants Δ*crtB* and Δ*crtI* was chosen to test whether *crtB2* and/or *crtI2-1/2* encode functional enzymes. Overexpression of *crtB2* almost completely complemented the *crtB* deletion and as HPLC analysis of extracts from *C. glutamicum* Δ*crtB*(pEKEx3-*crtB2*) indicated accumulation of decaprenoxanthin *crtB2* encodes a functional phytoene synthase (Figure 2, Additional file 5: Figure S3). By contrast, overexpression of *crtI2-1/2* in *C. glutamicum* Δ*crtI* did not restore the wild-type phenotype while overexpression of *crtI* did. Furthermore, while combined expression of *crtB2* and *crtI* in *C. glutamicum* strain ΔΔ led to an accumulation of lycopene, the combined expression of *crtB2* and *crtI2-1/2* did not (Additional file 6: Figure S4). Thus, whereas no evidence for *crtI2-1/2* encoding a phytoene desaturase was found, *crtB2* encodes an enzyme active as phytoene synthase.

### Enhancing lycopene production by overexpression of carotenogenic genes in the lycopene accumulating strain *C. glutamicum* Δ*crtEb*

**Figure 4:**
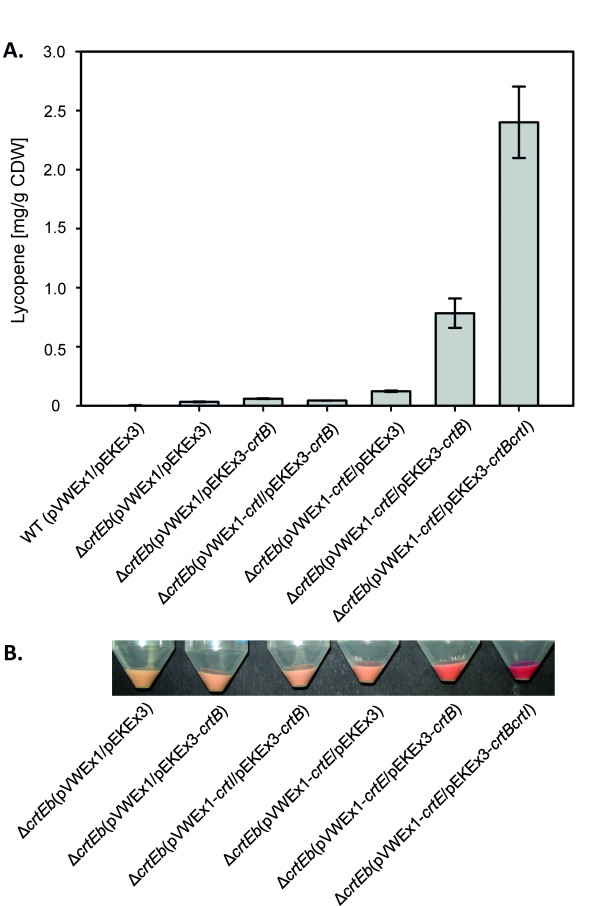
**Lycopene production in *C. glutamicum *Δ*crtEb *overexpressing carotenogenic genes.** (**A**) Cell pellets of cultures grown in glucose CGXII minimal medium after consumption of the carbon source. By the overexpression of the indicated carotenogenic genes the intensity of the red color was enhanced. (**B**) Lycopene concentrations of the cells depicted in A as determined by HPLC analyses of cell extracts.

The deletion of the gene *crtEb* entailed accumulation of lycopene to 0.03 ± 0.01 mg/g CDW in *C. glutamicum*. To enhance the production of lycopene we focused on improving conversion of GGPP to lycopene. Overexpression of the phytoene synthase gene *crtB* and/or the phytoene desaturase gene *crtI* in *C. glutamicum* Δ*crtEb* (Additional file 3: Table S1) was tested. Whereas *crtI* overexpression showed no effect on lycopene production (0.02 ± 0.01 mg/g CDW), it could be shown that lycopene accumulation was increased two-fold when *crtB* was overexpressed (0.06 ± 0.01 mg/g CDW, Figure 4). However, combined overexpression of both genes did not increase the lycopene content significantly (0.04 ± 0.01 mg/g CDW).

Besides overexpression of *crtB*, also overexpression of *crtE* which codes for the geranylgeranyl pyrophosphatase catalyzing the condensation of IPP and DMPP eventually leading to GGPP (Figure 2), increased lycopene production (Figure 4). As a consequence of overproduction of geranylgeranyl pyrophosphatase in *C. glutamicum* Δ*crtEb,* lycopene accumulated to four-fold higher concentrations (0.12 ± 0.01 mg/g CDW). The combined overexpression of *crtB* and *crtE* resulted in about 25 fold higher lycopene accumulation (0.8 ± 0.1 mg/g CDW, Figure 4) as compared to *C. glutamicum* Δ*crtEb*. The maximal lycopene concentration of 2.4 ± 0.3 mg/g CDW was achieved when all three enzymes, CrtE, CrtB and CrtI, were overproduced. Thus, after de-bottlenecking the CrtE reaction overexpression of *crtB* and *crtI* is beneficial for lycopene overproduction. The maximal lycopene accumulation was 80 fold higher than that of the empty vector control.

Lycopene production was associated with less biomass formation and slowed glucose consumption. In this regard the strain with the highest lycopene production, *C. glutamicum* Δ*crtEb*(pVWEx1*-crtE*/pEKEx3*-crtBI2)*, stood out. The cells reached the stationary phase after 32 h, exhausted glucose not before 54 h after inoculation and grew only to about half of the biomass concentration (3.7 ± 0.5 mg/ml CDW) as compared to the empty vector control (7.0 ± 0.2 mg/ml CDW).

## Discussion

The synthesis of C50 carotenoids occurs in a restricted number of bacterial species. Decaprenoxanthin is the most abundant one and it is the predominant carotenoid of the yellow *C. glutamicum*. The gene deletion and complementation analysis along with the pathway reconstruction in the multiple deletion strain *C. glutamicum* ΔΔ corroborates the previous elucidation of decaprenoxanthin biosynthesis in *C. glutamicum* based on transposon mutants of the strain MJ233C [16] and on heterologous expression of genes of the *crtE-cg0722-crtBIY*_*e*_*Y*_*f*_*Eb* cluster in the non-carotenogenic host *Escherichia. coli*[17]. Furthermore, we have analyzed a hitherto uncharacterized putative second carotenogenic gene cluster of *C. glutamicum*, *crtB2/crtI2-1/crtI2-2*, regarding the C50 carotenoid production. For the second phytoene synthase-like gene, *crtB2* (cg2672), annotated in the *C. glutamicum* genome [25] and postulated to be involved in the squalene synthesis [2], we provide evidence that *crtB2* indeed codes for a functional phytoene synthase. Hence, *C. glutamicum* possesses two functional phytoene synthases, CrtB and CrtB2. The two other open reading frames in the small *crt*-cluster are annotated as N- and C-terminal units of a second phytoene desaturase, but experimental confirmation of a phytoene desaturase function could not be obtained. Within the genus *Corynebacterium C. glutamicum* ATCC 13032 is the only species that possesses a second set of *crt* genes. The GC content of 54 to 58% of the second *crt* cluster is similar to the overall GC content of the genome, whereas that of the larger cluster is slightly lower. The genes of the two phytoene synthase paralogs only share 51% identity on the nucleotide level and mobile genetic elements such as IS-elements could not be detected in the vicinity of the two clusters arguing against recent duplication or horizontal gene transfer events.

All genome-sequenced corynebacterial species possess a *crtI* ortholog and most (except *C. variabile*) also possess a *crtB* ortholog, either clustered with *crtI* or elsewhere in the genome. The phylogeny of the *crtI* gene product reflects the phylogeny of the species. Only the highly related species *C. glutamicum* and *C. efficiens* exhibit all genes necessary to form C50 carotenoids. For corynebacterial species lacking some of the *crt* genes it remains to be shown if and which carotenoids are synthesized. On the other hand, *C. michiganense*[21], *C. erythrogenes*[22], *C. fascians*[23] and *C. poinsettiae*[24] are known to synthesize carotenoids, but their genome sequences are unknown.

In this study it could be shown that the genes of the carotenoid gene cluster of *C. glutamicum* ATCC 13032 *crtE-cg0722-crtBIY*_*e*_*Y*_*f*_*Eb* are co-transcribed. Similarly, also the second cluster is transciptionally organized as an operon. Transcriptional regulation of both operons has not yet been reported. The *in vivo* activity of the *crtB2* gene product appears low due either to low expression levels or to low catalytic activity as plasmid-borne overexpression was required to complement the phenotype of the deletion mutant lacking the paralog *crtB*. Currently, it remains unknown whether *crtB2* expression is affected by environmental stimuli and if/how the function of the two paralogs is regulated.

The potential of *C. glutamicum* for overproduction of carotenoids is to our knowledge described here for the first time. The interest in production of carotenoids, which find application in a wide variety of products due to their antioxidative properties and their colors, by cost-effective, environmentally friendly microbial fermentation processes is steadily increasing. The carotenogenic *C. glutamicum* is generally recognized as safe (GRAS), can readily be metabolically engineered and has been safely used in the million-ton-scale production of food-additives since more than 50 years [28]. Lycopene was chosen as a test carotenoid product as it may serve as a platform intermediate and as its red color serves as a simple read out. Lycopene is a commercial product obtained by fermentation with the fungus *Blakeslea trispora*[29] (Vitatene, Leon, Spain). Here we show that *C. glutamicum* overproduces lycopene if *crtEb* is deleted and that additional overexpression of the carotenogenic genes *crtE*, *crtB* and *crtI* boosted lycopene production 80 fold. The achieved lycopene concentration of 2.4 mg/g CDW is already comparable to that obtained with other popular biotechnological hosts like *E. coli,* for which e.g. a lycopene yield of 1.8 mg/g CDW was reported when the *crtE, crtB* and *crtI* genes of the plant pathogen *Pantoea ananatis* were overexpressed [20]. A higher lycopene concentration (6.6 mg/g CDW) could only be achieved in an *E. coli* strain overexpressing genes for IPP synthesis and carotenogenesis after a systematic screen identified three gene knockouts in the central carbon metabolism [30]. In *E. coli* harboring multiple modifications, i.e. carrying a plasmid with genes of the lycopene biosynthetic pathway (*crtE*, *crtB* and *crtI*) and a plasmid containing the entire heterologous MVA pathway as well as the IPP isomerase gene, *idi*, and overexpressing the endogenous *dxs* gene, a lycopene concentration of 6.8 ± 0.4 mg/g was obtained in batch culture [31]. Process engineering as fed-batch process with glycerol with 10 g/l glucose and 7.5 g/l arabinose boosted the lycopene concentration to 32 mg/g CDW [31].

The very good lycopene concentration obtained by *C. glutamicum* after engineering only the final three enzymatic steps of lycopene synthesis can likely be further enhanced by additional metabolic engineering of (a) IPP synthesis using the endogenous MEP pathway and/or the heterologous MVA pathway, (b) genome-based or computational approaches to identify target genes in the central metabolism or its regulation and (c) by process engineering using e.g. fed-batch protocols. Thus, *C. glutamicum* may serve as a suitable production host for lycopene and related carotenoids.

In addition, *C. glutamicum* is a natural producer of the relatively rare group of C50 carotenoids that feature strong antioxidative properties due to the multiple conjugated double bonds and the hydroxyl group [32-34]. The pharmaceutical potential of these C50 carotenoids is not yet well studied [35]. It is imaginable that decaprenoxanthin, its direct precursor flavuxanthin or the C50 carotenoid of *Micrococcus luteus*, sarcinaxanthin, could be of commercial interest. Notably, genes of *C. glutamicum* and of *M. luteus* have been used to engineer *E. coli* for the production of sarcinaxanthin [20]. Thus, the product range of structurally diverse C50 carotenoids could be accessible by engineered hosts including *C. glutamicum*.

## Conclusion

The genes of the carotenoid gene cluster of *C. glutamicum* ATCC 13032 *crtE-cg0722-crtBIY*_*e*_*Y*_*f*_*Eb* are co-transcribed and encode the enzymes involved in the biosynthesis of the C50 carotenoid decaprenoxanthin. An alternative, functionally active phytoene synthase is encoded in the *crtB2/crtI2-1/crtI2-2* operon leading to a certain degree of redundancy in carotenoid synthesis in *C. glutamicum*. The potential of *C. glutamicum* as production host for terpenoids in general was demonstrated by considerable lycopene production after engineering the terminal reactions leading to lycopene.

## Methods

### Bacterial strains, media and growth conditions

The strains and plasmids used in this work are listed in Additional file 3: Table S2. *C. glutamicum* ATCC 13032 was used as wild type (WT). Precultivation of *C. glutamicum* strains was performed in BHI or LB medium. For cultivation in CGXII medium [36] precultivated cells were washed once with CGXII medium without carbon source and inoculated to an initial OD_600_ of 1. Glucose was added as carbon and energy source to a concentration of 100 mM. Standard cultivations of *C. glutamicum* were performed at 30°C in a volume of 50 ml in 500 ml flasks with two baffles shaking at 120 rpm. The OD_600_ was measured in dilutions using a Shimadzu UV-1202 spectrophotometer (Duisburg, Germany). For cloning, *E. coli* DH5α was used as host and cultivated in LB medium at 37°C. When appropriate kanamycin or spectinomycin were added to concentrations of 25 μg/ml and 100 μg/ml, respectively. Gene expression was induced by adding 1 mM IPTG.

### Recombinant DNA work

Plasmids were constructed in *E. coli* DH5α from PCR-generated fragments (KOD, Novagen, Darmstadt, Germany) and isolated with the QIAprep spin miniprep kit (QIAGEN, Hilden, Germany). Oligonucleotides used in this study were obtained from Eurofins MWG Operon (Ebersberg, Germany) and are listed in Additional file 3: Table S1. Standard reactions like restriction, ligation and PCR were performed as described previously [37]. If applicable, PCR products were purified using the PCR purification kit or MinElute PCR purification kit (QIAGEN, Hilden, Germany). For transformation of *E. coli* the RbCl method was used [38] and *C. glutamicum* was transformed via electroporation [39] at 2.5 kV, 200 Ω and 25 μF. All cloned DNA fragments were shown to be correct by sequencing.

### Determination of the transcriptional start point of *crtE* and *crtB2*

Total RNA was isolated from an exponentially growing culture of *C. glutamicum* WT as described previously [40]. Purified RNA was analyzed by UV-spectrometry in regard to quantity and quality and was stored at −20°C until use. 2 μg of total RNA were used to perform 5’ rapid amplification of cDNA ends-PCR (5’ RACE_PCR) basically as described previously [41] with use of *crtE*-rv and *crtB2*-rv primers, respectively, for reverse transcription. Both, individual C tailing and A tailing were performed and analyzed. RACE_PCR was performed with primers *crtE*-RACE and *crtB2*-RACE and either OligoT or OligoG. Sequencing of the generated PCR fragments was accomplished using the suitable RACE primers and gave identical results for C tailing and A tailing reactions.

### Reverse transcription (RT) for the analysis of transcription units

Total RNA was isolated from an exponentially growing culture of *C. glutamicum* WT as described previously [40]. Purified RNA was analyzed by UV-spectrometry in regard to quantity and quality and was stored at −20°C until use. 2 μg of total RNA were used to perform reverse transcription to generate cDNA that was subsequently used as template for PCRs applying primer that bind at adjacent genes. The reverse transcription reactions were performed using SuperScript™ II reverse transcriptase (Invitrogen, Karlsruhe, Germany), and the remaining RNA was removed by the use of RNase H (MBI Fermentas GmbH, St. Leon-Rot).

### Overexpression of carotenogenic genes from *C. glutamicum*

Plasmids harboring a carotenogenic gene allowed its IPTG-inducible overexpression and were based on pEKEx3 [42] or pVWEx1 [43], respectively. Amplification of a carotenogenic gene by polymerase chain reaction (PCR) from genomic DNA of *C. glutamicum* WT, which was prepared as described [44], was carried out using the respective primers (Additional file 3: Table S1). The amplified products were cloned into the appropriately restricted pEKEx3 or pVWEx1 plasmid DNA.

### Deletion of carotenogenic genes in *C. glutamicum* WT

For deletion of a carotenogenic gene*,* the suicide vector pK19*mobsacB* was used [36]. Genomic regions flanking a carotenogenic gene were amplified from genomic DNA of *C. glutamicum* WT using the respective primer pairs A/B and C/D as indicated in Additional file 3: Table S1. The PCR products were purified and linked by crossover PCR using the respective primer pair A/D (Additional file 3: Table S1). The either *Sma*I or *Bam*HI restricted purified PCR product was cloned into pK19*mobsacB* resulting in the construction of the respective deletion vector (Additional file 3: Table S1). Targeted deletion of a carotenogenic gene via two-step homologous recombination using the respective deletion vector was carried out as described previously [26]. For the first recombination event integration of the vector into one of the flanking regions was selected via kanamycin resistance. Integration of the deletion vector into the genome results in a sucrose sensitivity because of the *sacB* gene product levansucrase. Selection for clones that have excised the deletion vector in a second recombination event was carried out via sucrose-resistance. Deletion of a carotenogenic gene was verified by PCR analysis of the constructed mutant using the respective primer pair E/F (Additional file 3: Table S1).

### Extraction of carotenoids from bacterial cell cultures

To extract carotenoids from the *C. glutamicum* strains 20 ml aliquots of the cell cultures were centrifuged at 10,000 × g for 15 min, and the pellets were washed with deionized H_2_O. The pigments were extracted with 10 ml methanol:acetone mixture (7:3) at 60°C for 80 min with thorough vortexing every 20 min. When necessary, several extraction cycles were performed to remove all visible colors from the cell pellet.

### Analysis of carotenoids

The extraction mixture was centrifuged 10,000 × g for 15 min and the methanol supernatant was transferred to a new tube. The absorption spectra of the various ex-tracts were measured at wavelengths between 400 and 800 nm using the UV-1202 spectrophotometer (Shimadzu, Duisburg, Germany). High performance liquid chromatography (HPLC) analyses of the *C. glutamicum* extracts were performed on an Agilent 1200 series HPLC system (Agilent Technologies Sales & Services GmbH & Co. KG, Waldbronn), including a diode array detector (DAD) for UV/visible (Vis) spectrum recording. Quantification of carotenoids was performed using the extracted wavelength chromatogram at peak λ_max_, 450 nm for decaprenoxanthin and carotenoids with corresponding UV/Vis profiles and 470 nm for lycopene and corresponding carotenoids. Lycopene from tomato (Sigma, Steinheim, Germany) was used as standard. It was dissolved in chloroform according to its solubility and diluted in methanol. The HPLC protocol comprised isocratic elution for 25 min using a flow rate of 1.4 ml/min, a mobile phase composition of methanol/methyl tert-butyl ether/ethyl acetate (5:4:1) and a column system consisting of a precolumn (10x4 mm MultoHigh 100 RP18-5, CS Chromatographie Service GmbH, Langerwehe, Germany) and a main column (ProntoSIL 200–5 C30, 250x4 mm, CS Chromatographie Service GmbH, Langerwehe, Germany). 100 μl extract was injected.

### Computational analysis

To compare the *crt* genes from *C. glutamicum* ATCC 13032 with other sequenced corynebacteria, the amino acid sequences of the respective genes were obtained from CoryneRegNet database (http://www.coryneregnet.de/). Sequence comparisons were carried out using BLASTP (http://www.ncbi.nlm.nih.gov/blast/Blast.cgi, [45]) and CLUSTALW [46] and the identification of potential orthologs and paralogs to *C. glutamicum* ATCC 13032 proteins was achieved by pairwise reciprocal BLAST analysis [47]. Species names, gene identifier and accession numbers are given in Additional file 1: Table S2. A phylogenetic tree was constructed using the neighbor joining method [48] with 1,000 bootstrap replicates.

## Abbreviations

DAD: Diode array detector; DMPP: Dimethylallyl pyrophosphate; EV: Empty vector; FPP: Farnesyl pyrophosphate; GAP: Glyceraldehyde 3-phosphate; GGPP: Geranylgeranyl pyrophosphate; GPP: Geranyl pyrophosphate; HPLC: High performance liquid chromatography; IPP: Isopentenyl pyrophosphate; IPTG: Isopropyl β-D-1-thiogalactopyranoside; MEP: Methylerythritol phosphate; MVA: Mevalonate; RACE: Rapid amplification of cDNA ends; RT: Reverse transcription; TSP: Transcriptional start point; WT: Wild type.

## Competing interests

The authors do not declare competing interests.

## Authors’ contributions

All authors contributed to designing the study. SAEH constructed and characterized the recombinant strains. VFW and PPW supervised the experiments. SAEH and PPW were responsible for the draft of the manuscript. All authors contributed to writing and approved the final manuscript.

## Supplementary Material

Additional file 1 **Table S1.** Comparison of the *crt* genes from different corynebacteria, for which genome sequence information is available in the database (NCBI). The rows show the gene identifier (top) and accession number (middle) for each *crt* gene of the corresponding species and the amino acid identity to the respective *crt* gene product from *C. glutamicum* ATCC 13032 (bottom). Click here for file

Additional file 2 **Figure S1.** Phylogenetic tree of phytoene desaturase from different corynebacteria. Numbers at the nodes represent bootstrap values. Gene identifiers are given in Additional file 1: Table S2. Click here for file

Additional file 3 **Table S2. **Bacterial strains, plasmids and oligonucleotides [38,40,47,49,50]. Click here for file

Additional file 4 **Figure S2.** HPLC chromatograms of carotenoids extracted from *C. glutamicum* Δ*crtB* strains (A) and Δ*crtI* (B).Detection by absorption at 470 nm. (A) Elution profiles of carotenoids extracted from *C. glutamicum* WT (blue), Δ*crtB*(pEKEx3) (red), Δ*crtB*(pEKEx3-*crtB*) (green), Δ*crtB*(pEKEx3-*crtB2*) (pink). (B) Elution profiles of carotenoids extracted from *C. glutamicum* WT (blue), Δ*crtI*(pEKEx3) (red), Δ*crtI*(pEKEx3-*crtI*) (green), Δ*crtI*(pEKEx3-*crtI2-1/2*) (pink). Click here for file

Additional file 5 **Figure S3.** Absorption spectra of cell extracts of *C. glutamicum* WT and *crt* deletion strains. The extract of the strains *C. glutamicum* Δ*crtEb* (black line) and Δ*crtY* (grey line) show an additional absorption maximum at about 500 nm compared to the wild type (red line). *C. glutamicum* Δ*crtB* (dotted line) and Δ*crtI* (dashed line) show no absorption. Click here for file

Additional file 6 **Figure S4.** HPLC elution profiles of carotenoids extracted from *C. glutamicum* ΔΔ strains. Detection by absorption at 470 nm. (A) Elution profiles of carotenoids extracted from *C. glutamicum* ΔΔ(pEKEx3/pVWEx1) (blue), ΔΔ(pEKEx3-*crtB*/pVWEx1-*crtI*) (red), ΔΔ(pEKEx3-*crtB2*/pVWEx1-*crtI*) (green). The red and green chromatograms show the accumulation of carotenoids including lycopene (L) which elutes after about 18 min. (B) Elution profiles of carotenoids extracted from *C. glutamicum* ΔΔ(pEKEx3/pVWEx1) (blue) and ΔΔ(pEKEx3-*crtI2-1*/2/pVWEx1-*crtB2*) (red). Click here for file
