# Supplementary material for: Carotenoid biosynthesis and overproduction in Corynebacterium glutamicum
Source: BMC Microbiol. 2012 Sep 10;12:198. doi: 10.1186/1471-2180-12-198 (PMC3598387; doi:10.1186/1471-2180-12-198)
Supplement: Additional file 1 — Table S1. Comparison of the crt genes from different corynebacteria, for which genome sequence information is available in the database (NCBI). The rows show the gene identifier (top) and accession number (middle) for each crt gene of the corresponding species and the amino acid identity to the respective crt gene product from C. glutamicum ATCC 13032 (bottom). [file 1471-2180-12-198-S1.docx]

Supplementary Table 2: Comparison of the *crt* genes from different corynebacteria, for which genome sequence information is available in the database (NCBI). The rows show the gene identifier (top) and accession number (middle) for each *crt* gene of the corresponding species and the amino acid identity to the respective *crt* gene product from *C. glutamicum* ATCC 13032 (bottom).

| **Organism** | ***crtE*** | ***mmpL3* like** | ***crtB*** | ***crtI*** | ***crtYe*** | ***crtYf*** | ***crtEb*** | ***crtB2*** | ***ctrI2-1/2*** |
| --- | --- | --- | --- | --- | --- | --- | --- | --- | --- |
| *C. glutamicum*  ATCC 13032 | cg0723  YP_224919 | cg0722  YP_224918 | cg0721  YP_224917 | cg0720  YP_224916 | cg0719  YP_224915 | cg0718  YP_224914 | cg0717  YP_224913 | cg2672  YP_226675 | cg2670/cg2668  YP_226674/ YP_226673 |
| *C. glutamcium* R | cgR_0745  YP_001137619.1  352/372 (95%) | cgR_0744  YP_001137618.1  704/730 (96%) | cgR_0743  YP_001137617.1  289/304 (98%) | cgR_0742  YP_001137616.1  541/548 (99%) | Not annotated | Not annotated | cgR_0740  YP_001137613.1  284/287 (99%) |  |  |
| *C. glutamicum* S9114 | CgS9114_02583  EGV41659.1  290/307 (94%) | CgS9114_02578  EGV41658.1  695/712 (98%) | CgS9114_02573  EGV41657.1  297/304 (98%) | CgS9114_02568  EGV41656.1  534/548 (97%) | CgS9114_02563  EGV41655.1  125/132 (95%) | CgS9114_02558  EGV41654.1  122/130 (94%) | CgS9114_02553  EGV41653.1  281/287 (98%) |  |  |
| *C. glutamicum*  ATCC 14067 | KIQ_04850  EHE84235.1  290/307 (94%) | KIQ_04845  EHE84234.1  696/712 (98%) | KIQ_04840  EHE84233.1  297/304 (98%) | KIQ_04835  EHE84232.1  534/548 (97%) | KIQ_04830  EHE84231.1  141/149 (95%) | KIQ_04825  EHE84230.1  119/130 (92%) | KIQ_04820  EHE84229.1  278/287 (97%) |  |  |
| *C. efficiens* YS-314 | CE0643  NP_737253.1  249/374 (67%) | CE0642  NP_737252.1  570/733 (78%) | CE0641  NP_737251.1  206/295 (70%) | CE0640  NP_737250.1  419/524 (80%) | CE0639  NP_737249.1  85/136 (63%) | CE0638  NP_737248.1  67/107 (63%) | CE0637  NP_737247.1  213/287 (74%) |  |  |
| *C.lipophiloflavum* |  |  | HMPREF0298_0517  ZP_03978253.1  148/277 (53%) | HMPREF0298_0518  ZP_03978254.1  297/512 (58%) | HMPREF0298_0519  ZP_03978255.1  52/95 (55%) | HMPREF0298_0520  ZP_03978256.1  36/66 (55%) | HMPREF0298_0521  ZP_03978257.1  168/275 (61%) |  |  |
| *C. genitalium*  ATCC 33030 |  |  | HMPREF0291_10934  ZP_07090363.1  139/284 (49%) | HMPREF0291_10935  ZP_07090364.1  305/522 (58%) | HMPREF0291_10936  ZP_07090365.1  52/95 (55%) | HMPREF0291_10937  ZP_07090366.1  41/91 (45%) |  |  |  |
| *C. matruchotii*  ATCC 33806 (*et al.*) |  |  | CORMATOL_00647  ZP_03709832.1  140/265 (53%) | CORMATOL_00646  ZP_03709831.1  286/515 (56%) |  |  |  |  |  |
| *C. diphtheriae*  NCTC 13129 (*et al.*) |  |  | DIP1870  NP_940207.1  147/282 (52%) | DIP1871  NP_940208.1  279/511 (55%) |  |  |  |  |  |
| *C. accolens* ATCC 49725 (*et al.*) |  |  | HMPREF0276_0678  ZP_03932124.1  143/275 (52%) | HMPREF0276_0677  ZP_03932123.1  310/533 (58%) |  |  |  |  |  |
| *C. aurimucosum* | cauri_1683 (*idsA)*  YP_002835214.1 |  | cauri_2128  YP_002835659.1  142/279 (51%) | cauri_2129  YP_002835660.1  295/508 (58%) |  |  |  |  |  |
| *C. striatum* ATCC 6940 |  |  | HMPREF0308_0241  ZP_03934020.1  150/295 (51%) | HMPREF0308_0242  ZP_03934021.1  305/525 (58%) |  |  |  |  |  |
| *C. pseudogenitalium* ATCC 33035 |  |  | HMPREF0305_10930  ZP_07713995.1  139/277 (50%) | HMPREF0305_10929  ZP_07713994.1  316/518 (61%) |  |  |  |  |  |
| *C. tuberculostearicum* SK141 |  |  | CORTU0001_0166  ZP_05366946.1  138/277 (50%) | CORTU0001_0165  ZP_05366945.1  314/518 (61%) |  |  |  |  |  |
| *C. pseudotuberculosis* FRC41 (*et al.*) |  |  | cpfrc_02080  YP_003784480.1  148/298 (50%) | cpfrc_02081  YP_003784481.1  316/543 (58%) |  |  |  |  |  |
| *C. ulcerans* BR-AD22  (*et al.*) |  |  | CULC22_02324  YP_004630945.1  147/297 (49%) | CULC22_02325  YP_004630946.1  315/540 (58%) |  |  |  |  |  |
| *C. ammoniagenes*  DSM 20306 |  |  | HMPREF0281_01477  ZP_06837527.1  143/291 (49%) | HMPREF0281_01476  ZP_06837526.1  297/552 (54%) |  |  |  |  |  |
| *C. casei* UCMA 3821 |  |  | CCAS_08445 ZP_09156874.1  142/292 (49%) | CCAS_08450 ZP_09156875.1  295/573 (51%) |  |  |  |  |  |
| *C. amycolatum* SK46 |  |  | CORAM0001_1124 ZP_03393472.1  61/200 (31%) | CORAM0001_1122 ZP_03393431.1  139/568 (24%) |  |  |  |  |  |
| *C. nuruki* S6-4 |  |  | CnurS_010100005359 ZP_09128268.1  133/281 (47%) | CnurS_010100005354 ZP_09128267.1  294/519 (57%) |  |  |  |  |  |
| *C. glucuronolyticum* ATCC 51867 (*et al.*) |  |  | HMPREF0294_0763 ZP_03917929.1  127/259 (49%) | HMPREF0294_1539 ZP_03918705.1  255/523 (49%) |  |  |  |  |  |
| *C. kroppenstedtii* DSM 44385 | ckrop_0731 (*idsA*)  YP_002906034.1 |  | ckrop_1332 YP_002906618.1  129/294 (44%) | ckrop_0730 YP_002906033.1  142/577 (25%) |  |  |  |  |  |
| *C. urealyticum* DSM 7109 |  |  | cur_1398 YP_001800792.1  125/294 (43%) | cur_1220 YP_001800614.1  144/564 (26%) |  |  |  |  |  |
| *C. jeikeium* K411 (*et al.*) |  |  | jk051  YP_250291.1  119/284 (42%) | jk0735  YP_250515.1  135/576 (23%) |  |  |  |  |  |
| *C. resistens* DSM 45100 |  |  | CRES_0603 YP_004605128.1  115/289 (40%) | CRES_0778 YP_004605298.1  145/541 (27%) |  |  |  |  |  |
| *C. bovis* DSM 20582 |  |  | CbovD2_010100005647 ZP_08517030.1  104/237 (44%) | CbovD2_010100002087 ZP_08516328.1  291/508 (57%) |  |  |  |  |  |
| *C. variabile* DSM 44702 |  |  |  | CVAR_0536 YP_004758959.1  310/530 (58%) |  |  |  |  |  |
