# Supplementary material for: Carotenoid biosynthesis and overproduction in Corynebacterium glutamicum
Source: BMC Microbiol. 2012 Sep 10;12:198. doi: 10.1186/1471-2180-12-198 (PMC3598387; doi:10.1186/1471-2180-12-198)
Supplement: Additional file 3 — Table S2. Bacterial strains, plasmids and oligonucleotides [38,40,47,49,50]. [file 1471-2180-12-198-S3.docx]

Supplementary table 1: **Bacterial strains, plasmids and oligonucleotides**

| **Strain, plasmid or oligo-nucleotide** | | **Relevant characteristics or sequence** | | **Source or reference** |
| --- | --- | --- | --- | --- |
| ***E. coli* strains** | | | | |
|  | DH5α | F^-^ *thi*-1 *endA1* *hsdR17*(r^-^ m^-^) *supE44* Δ*lacU169* (φ80*lacZ*ΔM15) *recA1 gyrA96 relA1* | | [35] |
|  |  |  | |  |
| ***C. glutamicum* strains** | | | | |
|  | WT^a^ | ATCC 13032 | | [50] |
|  | Δ*crtB* | *crtB2* deletion mutant of *C. glutamicum* WT | | this work |
|  | Δ*crtI* | *crtI2* deletion mutant of *C. glutamicum* WT | | this work |
|  | Δ*crtEb* | *crtEb* deletion mutant of *C. glutamicum* WT | | this work |
|  | Δ*crtY* | *crtY_e_ Y_f_* deletion mutant of *C. glutamicum* WT | | this work |
|  | Δ*crtB* | *crtB* deletion mutant of *C. glutamicum* WT | | this work |
|  | Δ*crtB2I2-1/2* | *crtB2I2-1I2-2* deletion mutant of *C. glutamicum* WT | | this work |
|  | Δ*crtB2I2-1/2*Δ*crtI* | *crtB2I2-1I2-2* and *crtI* deletion mutant of *C. glutamicum* WT | | this work |
|  | Δ*crtB2I2-1/2*Δ*crtB* | *crtB2I2-1I2-2* and *crtB* deletion mutant of *C. glutamicum* WT | | this work |
|  | ΔΔ | *crtB2I2-1I2-2* and *crtBIYEb* deletion mutant of *C. glutamicum* WT | | this work |
|  |  |  | |  |
| **Plasmids** | | | | |
|  | pK19*mobsacB* | Km^R^; *E. coli*/*C. glutamicum* shuttle vector for construction of insertion and deletion mutants in *C. glutamicum* (pK18 *oriV_Ec_ sacB lacZ*α) | | [45] |
|  | pK19*mobsacB*-Δ*crtB* | pK19*mobsacB* with a *crtB* deletion construct | | this work |
|  | pK19*mobsacB*-Δ*crtI* | pK19*mobsacB* with a *crtI* deletion construct | | this work |
|  | pK19*mobsacB*-Δ*crtEb* | pK19*mobsacB* with a *crtEb* deletion construct | | this work |
|  | pK19*mobsacB*-Δ*crtY* | pK19*mobsacB* with a *crtY_e_Y_f_* deletion construct | | this work |
|  | pK19*mobsacB*-Δ*crtB2* | pK19*mobsacB* with a *crtB* deletion construct | | this work |
|  | pK19*mobsacB*-Δ Δ*crtB2I2-1/2* | pK19*mobsacB* with a Δ*crtB2I2-1I2-2* deletion construct | | this work |
|  | pK19*mobsacB*-Δ*crtBIYEb* | pK19*mobsacB* with a deletion construct for the entire *crtBIYEb* fragment | | this work |
|  | pVWEx1 | Km^R^; *E. coli*/*C. glutamicum* shuttle vector for regulated gene expression (P_tac_, *lacI*^q^, pCG1 *oriV_Cg_*) | | [40] |
|  | pVWEx1-*crtB2* | pVWEx1 derivative for IPTG-inducible expression of *crtB2* from *C. glutamicum* containing an artificial ribosome binding site | | this work |
|  | pVWEx1-*crtI* | pVWEx1 derivative for IPTG-inducible expression of *crtI* from *C. glutamicum* containing an artificial ribosome binding site | | this work |
|  | pVWEx1-*crtEb* | pVWEx1 derivative for IPTG-inducible expression of *crtEb* from *C. glutamicum* containing an artificial ribosome binding site | | this work |
|  | pVWEx1-*crtBI* | pVWEx1 derivative for IPTG-inducible expression of *crtBI* from *C. glutamicum* containing a single artificial ribosome binding site in front of *crtB2* | | this work |
|  | pVWEx1-*crtE* | pVWEx1 derivative for IPTG-inducible expression of two tandem copies of *crtE* from *C. glutamicum* containing artificial ribosome binding sites | | this work |
|  | pEKEx3 | Spec^R^; *E. coli*/*C. glutamicum* shuttle vector for regulated gene expression (P_tac_, *lacI*^q^, pBL1 *oriV_Cg_*) | | [39] |
|  | pEKEx3-*crtB* | pEKEx3 derivative for IPTG-inducible expression of *crtB* from *C. glutamicum* containing an artificial ribosome binding site | | this work |
|  | pEKEx3-*crtI* | pEKEx3 derivative for IPTG-inducible expression of *crtI*  from *C. glutamicum* containing an artificial ribosome binding site | | this work |
|  | pEKEx3-*crtEb* | pEKEx3 derivative for IPTG-inducible expression of *crtEb* from *C. glutamicum* containing an artificial ribosome binding site | | this work |
|  | pEKEx3-*crtY* | pEKEx3 derivative for IPTG-inducible expression of *crtY_e_* and *crtY_f_* from *C. glutamicum* containing an artificial ribosome binding site | | this work |
|  | pEKEx3-*crtBI* | pEKEx3 derivative for IPTG-inducible expression of *crtBI* from *C. glutamicum* containing a single artificial ribosome binding site in front of *crtB2* | | this work |
|  | pEKEx3-*crtYEb* | pEKEx3 derivative for IPTG-inducible expression of *crtYEb* from *C. glutamicum* containing a single artificial ribosome binding site in front of *crtY* | | this work |
|  | pEKEx3-*crtB2* | pEKEx3 derivative for IPTG-inducible expression of *crtB2* from *C. glutamicum* containing an artificial ribosome binding site | | this work |
|  | pEKEx3-*crt2-1/2* | pEKEx3 derivative for IPTG-inducible expression of *crtI2-1I2-2* from *C. glutamicum* containing an artificial ribosome binding site | | this work |
|  |  |  |  | |
| **oligonucleotides** | | | | |
|  |  | **sequence (5’→3’)** | | **underlined nucleotide (position in NC003450)** |
|  | *crtB*-A ^c^ | AAA*CCCGGG*TTCAATCACGTTTTCAGTTTCCCA | |  |
|  | *crtB*-B ^d^ | ***CCCATCCACTAAACTTAAACA***AGGCGAATTTTGGTGTGTCAT | |  |
|  | *crtB*-C ^d^ | ***TGTTTAAGTTTAGTGGATGGG***AGCATCTACAGAAAGAATTCGTGA | |  |
|  | *crtB*-D ^c^ | AAASA*CCCGGG*TAGTTTTGCACCCGCGC | |  |
|  | *crtB*-E | CATGACCGCAACCTGATCA | |  |
|  | *crtB*-F | TGTAAAGAACGGGTCAGTAGAG | |  |
|  | *crtI*-A ^c^ | AAAA*CCCGGG*GGCTAGAGATTATGCAAAACGG | |  |
|  | *crtI*-B ^d^ | ***CCCATCCACTAAACTTAAACA***AGTTTTAGTCGAGACCTTCATCAC | |  |
|  | *crtI*-C ^d^ | ***TGTTTAAGTTTAGTGGATGGG***TCTCAAAAGACCTCATACGATCATTAA | |  |
|  | *crtI*-D ^c^ | AAAA*CCCGGG*AGTATCGCGCTGTGGTGT | |  |
|  | *crtI*-E | CATCAATGCGTAAGGACCTC | |  |
|  | *crtI*-F | CTGACAGCGGTGATTGGTAA | |  |
|  | *crtEb*-A ^c^ | AAAA*CCCGGG*ACTACCACTCCCGAGGTT | |  |
|  | *crtEb*-B ^d^ | ***CCCATCCACTAAACTTAAACA***TAGAATTAGTCTTATTTTTTCCATCAT | |  |
|  | *crtEb*-C ^d^ | ***TGTTTAAGTTTAGTGGATGGG***ACGATACTGCTAATAGCAATTCATCAGATATAA | |  |
|  | *crtEb*-D ^c^ | AAAA*CCCGGG*ATGTGTGGGAGGCTTCGC | |  |
|  | *crtEb*-E | GGAGACTCAGCGTTTATGTC | |  |
|  | *crtEb*-F | AAAACAATGCGCAGCGCA | |  |
|  | *crtY*-A ^e^ | AAAA*GGATCC*AGTCGGCTTCAGCATCC | |  |
|  | *crtY*-B ^d^ | ***CCCATCCACTAAACTTAAACA***TGAAATATCGATGATAGGGATCAA | |  |
|  | *crtY*-C ^d^ | ***TGTTTAAGTTTAGTGGATGGG***GAGCCAGAAAAGCCGTAG | |  |
|  | *crtY*-D ^e^ | AAAA*GGATCC*CTTTAAAACGCAATTTCGGTGC | |  |
|  | *crtY*-E | TTGCACCTGCTGGATACGAA | |  |
|  | *crtY*-F | ATCGCTGCTGAAGGAGATGT | |  |
|  | *crtB2*-A ^c^ | AAAA*CCCGGG*GTCAGTGCTGTCATCGGTAC | |  |
|  | *crtB2*-B ^d^ | ***CCCATCCACTAAACTTAAACA***ATCTTGCTGATCAGCCAC | |  |
|  | *crtB2*-C ^d^ | ***TGTTTAAGTTTAGTGGATGGG***TGGAAAGAAGTGTTTCAAAAATGA | |  |
|  | *crtB2*-D ^c^ | AAAA*CCCGGG*GGCCAGGTTGTAGACCTG | |  |
|  | *crtB2*-E | CGGACTTGATGCTGCAGC | |  |
|  | *crtB2*-F | TCGGTGTGGCTCATGAG | |  |
|  | *crtB2-*RACE | GCATGGATCACTGGATCGG | |  |
|  | *crtI2-1/2*-A ^c^ | AAAA*CCCGGGG*GCTAGAGATTATGCAAAACGG | |  |
|  | *crtI2*-B ^d^ | ***CCCATCCACTAAACTTAAACA***AGTTTTAGTCGAGACCTTCATCAC | |  |
|  | *crtI2*-C ^d^ | ***TGTTTAAGTTTAGTGGATGGG***TCTCAAAAGACCTCATACGATCATTAA | |  |
|  | *crtI2*-D ^c^ | AAAA*CCCGGG*AGTATCGCGCTGTGGTGT | |  |
|  | *crtI2*-E | CATCAATGCGTAAGGACCTC | |  |
|  | *crtI2*-F | CTGACAGCGGTGATTGGTAA | |  |
|  | *crtB* -*Pst*I-fw ^b,f^ | AAAA*CTGCAG***GAAAGGAGGCCCTTCAG**ATGACACACCAAAATTCGCC | | 636636 |
|  | *crtB* -*BamH*I-fw ^b,f^ | AAAA*GGATCC***GAAAGGAGGCCCTTCAG**ATGACACACCAAAATTCGCC | | 636636 |
|  | *crtB* -*Bam*HI-rv ^b,f^ | AAAA*GGATCC*TCACGAATTCTTTCTGTAGATGC | |  |
|  | *crtB2*-*Pst*I-fw ^b,f^ | TTTT*CTGCAG***GAAAGGAGGCCCTTCAG**GTGGCTGATCAGCAAGATTT | | 2545516 |
|  | *crtB2*-*Bam*HI-rv ^b,f^ | TTTT*GGATCC*TCATTTTTGAAACACTTCTTTCCA | |  |
|  | *crtI* -*Bam*HI-fw ^b,f^ | TTTT*GGATCC***GAAAGGAGGCCCTTCAG**GTGATGAAGGTCTCGACTAA | | 634990 |
|  | *crtI* -*Eco*RI-rv ^b,f^ | TTTT*GAATTC*TTAATGATCGTATGAGGTCTTTTG | |  |
|  | *crtI* -*Bam*HI-rv ^b,f^ | TTTT*GGATCC*TTAATGATCGTATGAGGTCTTTTG | |  |
|  | *crtI2*-*Bam*HI-fw ^b,f^ | TTTT*GGATCC***GAAAGGAGGCCCTTCAG**ATGACAAAAGCAGTGGTCAT | | 2544332 |
|  | *crtI2*-*Sac*I-rv ^b,f^ | TTTT*GAGCTC*TTAAGTCCGATCCACACTGT | |  |
|  | *crtY*-*Bam*HI-fw ^b,f^ | AAAA*GGATCC***GAAAGGAGGCCCTTCAG**TTGATCCCTATCATCGATATTTCA | | 634537 |
|  | *crtY*-*Eco*RI-fw ^b,f^ | AAAA*GAATTC***GAAAGGAGGCCCTTCAG**TTGATCCCTATCATCGATATTTCA | | 634537 |
|  | *crtY*-*Eco*RI-rv ^b,f^ | AAAA*GAATTC*CTACGGCTTTTCTGGCTCA | |  |
|  | *crtEb-Pst*I-fw ^b,f^ | AAAA*CTGCAG***GAAAGGAGGCCCTTCAG**ATGATGGAAAAAATAAGACTAATTC | | 633282 |
|  | *crtEb-Eco*RI-rv ^b,f^ | AAAA*GAATTC*TTATATCTGATGAATTGCTATTAGC | |  |
|  | *crtEb-Pst*I-rv ^b,f^ | AAAA*CTGCAG*TTATATCTGATGAATTGCTATTAGC | |  |
|  | *crtE-Pst*I-fw ^b,f^ | AAAA*CTGCAG***GAAAGGAGGCCCTTCAG**ATGGACAATGGCATGACAATC | | 640893 |
|  | *crtE-Pst*I-rv ^b,f^ | AAAA*CTGCAG*CTAAGATTTGCGGCTGGC | |  |
|  | *crtE*-RACE | CAGGTGTGCGATGCATGA | |  |
|  | pVWEx-fw | CATCATAACGGTTCTGGC | |  |
|  | pVWEx-rv | ATCTTCTCTCATCCGCCA | |  |
|  | M13 fw | CACAGCGGGAGTGCCTATTGTTTTG | |  |
|  | M13 rv | CAGCGATGATCACTTCTGGCTC | |  |
|  | OligoT | GACCACGCGTATCGATGTCGACTTTTTTTTTTTTTTTTTT | |  |
|  | OligoG | GACCACGCGTATCGATGTCGACGGGGGGGGGGGGGGGGGG | |  |
| ^a^ WT, wild type | | | | |
| ^b^sequence in bold: artificial ribosome binding site | | | | |
| ^c^sequence in italic: *Sma*I restriction site | | | | |
| ^d^sequence in bold and italic: linker sequence for hybridization | | | | |
| ^e^sequence in italic: *Bam*HI restriction site | | | | |
| ^f^sequence in italic: restriction site | | | | |
