# Supplementary figures and images for: Carotenoid biosynthesis and overproduction in Corynebacterium glutamicum
Source: BMC Microbiol. 2012 Sep 10;12:198. doi: 10.1186/1471-2180-12-198 (PMC3598387; doi:10.1186/1471-2180-12-198)

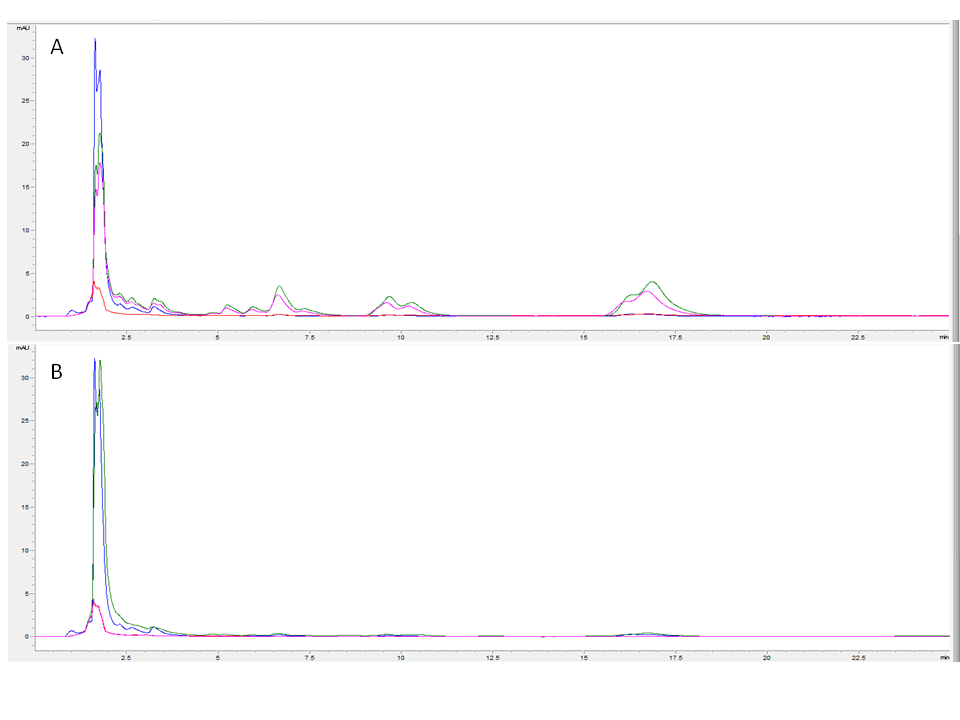

Supplement: Additional file 4 — Figure S2. HPLC chromatograms of carotenoids extracted from C. glutamicum ΔcrtB strains (A) and ΔcrtI (B).Detection by absorption at 470 nm. (A) Elution profiles of carotenoids extracted from C. glutamicum WT (blue), ΔcrtB(pEKEx3) (red), ΔcrtB(pEKEx3-crtB) (green), ΔcrtB(pEKEx3-crtB2) (pink). (B) Elution profiles of carotenoids extracted from C. glutamicum WT (blue), ΔcrtI(pEKEx3) (red), ΔcrtI(pEKEx3-crtI) (green), ΔcrtI(pEKEx3-crtI2-1/2) (pink). [file 1471-2180-12-198-S4.png]

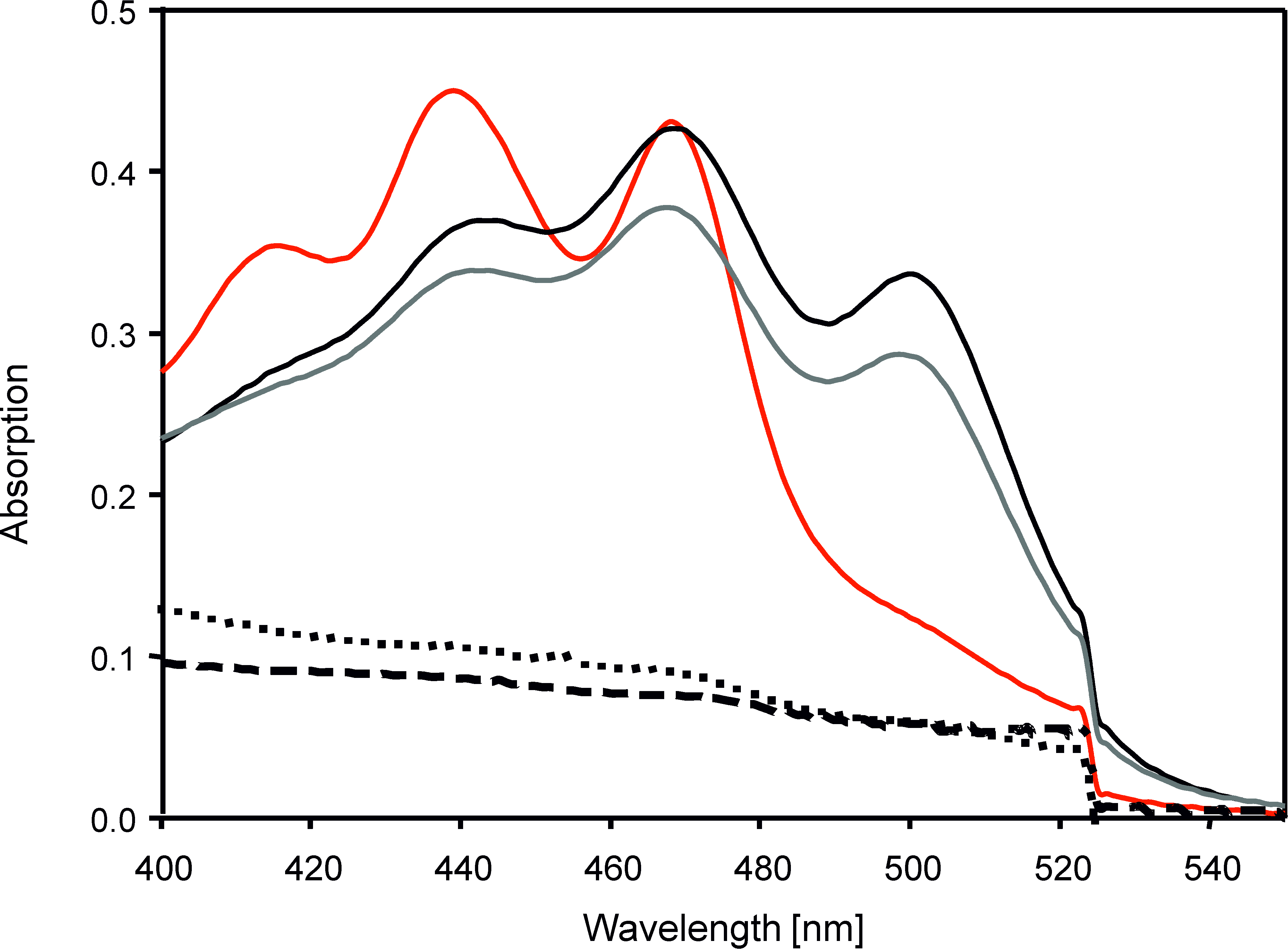

Supplement: Additional file 5 — Figure S3. Absorption spectra of cell extracts of C. glutamicum WT and crt deletion strains. The extract of the strains C. glutamicum ΔcrtEb (black line) and ΔcrtY (grey line) show an additional absorption maximum at about 500 nm compared to the wild type (red line). C. glutamicum ΔcrtB (dotted line) and ΔcrtI (dashed line) show no absorption. [file 1471-2180-12-198-S5.tiff]

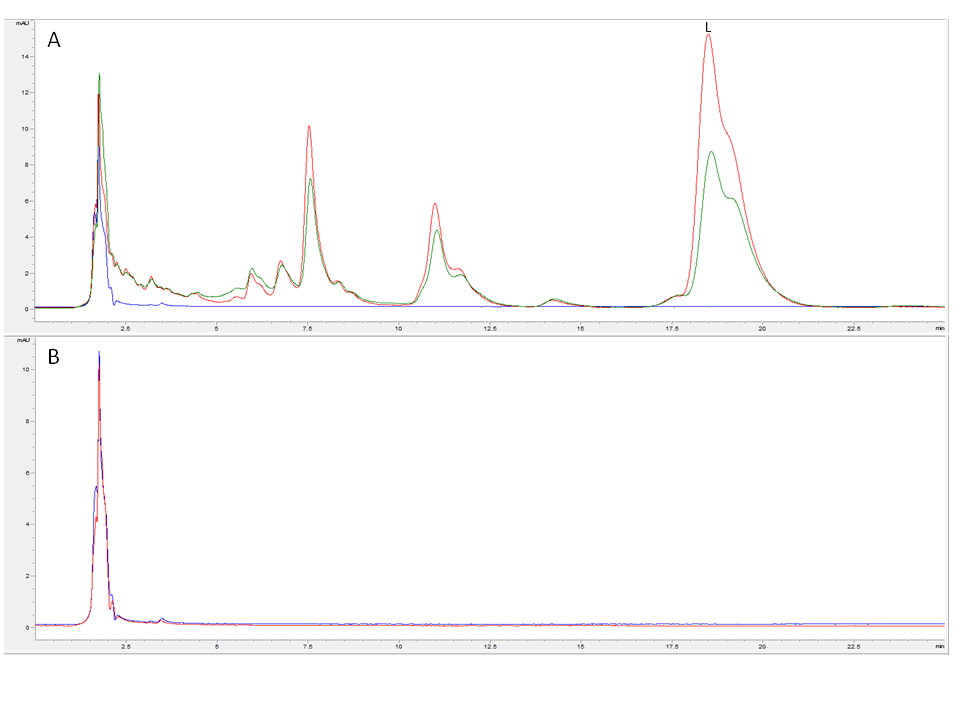

Supplement: Additional file 6 — Figure S4. HPLC elution profiles of carotenoids extracted from C. glutamicum ΔΔ strains. Detection by absorption at 470 nm. (A) Elution profiles of carotenoids extracted from C. glutamicum ΔΔ(pEKEx3/pVWEx1) (blue), ΔΔ(pEKEx3-crtB/pVWEx1-crtI) (red), ΔΔ(pEKEx3-crtB2/pVWEx1-crtI) (green). The red and green chromatograms show the accumulation of carotenoids including lycopene (L) which elutes after about 18 min. (B) Elution profiles of carotenoids extracted from C. glutamicum ΔΔ(pEKEx3/pVWEx1) (blue) and ΔΔ(pEKEx3-crtI2-1/2/pVWEx1-crtB2) (red). [file 1471-2180-12-198-S6.png]
